# Prevalence, incidence and survival of heart failure: a systematic review

**DOI:** 10.1136/heartjnl-2021-320131

**Published:** 2022-01-18

**Authors:** Sophia Emmons-Bell, Catherine Johnson, Gregory Roth

**Affiliations:** 1 Institute for Health Metrics and Evaluation, University of Washington, Seattle, Washington, USA; 2 Division of Cardiology, Department of Medicine, University of Washington, Seattle, Washington, USA

**Keywords:** heart failure, Global Burden of Disease

## Abstract

Studies of the epidemiology of heart failure in the general population can inform assessments of disease burden, research, public health policy and health system care delivery. We performed a systematic review of prevalence, incidence and survival for all available population-representative studies to inform the Global Burden of Disease 2020. We examined population-based studies published between 1990 and 2020 using structured review methods and database search strings. Studies were sought in which heart failure was defined by clinical diagnosis using structured criteria such as the Framingham or European Society of Cardiology criteria, with studies using alternate case definitions identified for comparison. Study results were extracted with descriptive characteristics including age range, location and case definition. Search strings identified 42 360 studies over a 30-year period, of which 790 were selected for full-text review and 125 met criteria for inclusion. 45 sources reported estimates of prevalence, 41 of incidence and 58 of mortality. Prevalence ranged from 0.2%, in a Hong Kong study of hospitalised heart failure patients in 1997, to 17.7%, in a US study of Medicare beneficiaries aged 65+ from 2002 to 2013. Collapsed estimates of incidence ranged from 0.1%, in the EPidémiologie de l’Insuffisance Cardiaque Avancée en Lorraine (EPICAL) study of acute heart failure in France among those aged 20–80 years in 1994, to 4.3%, in a US study of Medicare beneficiaries 65+ from 1994 to 2003. One-year heart failure case fatality ranged from 4% to 45% with an average of 33% overall and 24% for studies across all adult ages. Diagnostic criteria, case ascertainment strategy and demographic breakdown varied widely between studies. Prevalence, incidence and survival for heart failure varied widely across countries and studies, reflecting a range of study design. Heart failure remains a high prevalence disease among older adults with a high risk of death at 1 year.

## Introduction

Studies of the epidemiology of heart failure in the general population can inform assessments of disease burden, research, public health policy and health system care delivery. Past investigations of the occurrence of heart failure in the community have most often been performed in the high-income world, however prevalence is projected to rise in low-income and middle-income countries as populations age and the burden of heart failure risk factors such as elevated blood pressure increases in the coming decades.^1^ Heart failure is also likely to confer significant economic burden to individuals and health systems.^2^

The Global Burden of Disease (GBD) study produces comprehensive and comparable estimates of disease burden for 370 causes for 204 countries and territories from 1990 to 2020, using disease modelling methods.^3^ Regular reviews of published scientific studies are performed to identify data on disease burden, including for heart failure. A focus of this review is the systematic identification of all available data from all countries, with care taken to account for stratification by age and sex, and sought over long timeframes to capture secular trends. Particular attention is paid to variation in disease case definitions and how this may influence observations. Previous reviews by other groups have focused on subtypes of heart failure, specific age groups^4 5^ specific geographic regions^6–11^ or were restricted to prevalence rates only. To date, no review has included prevalence, incidence and rates of survival, covered all geographic regions and included studies from 1990 to the current day. Here, we report the results of such a systematic review identifying data sources to inform the GBD 2020 study estimates of heart failure.

## Methods

Our review was designed to address specific challenges in the reporting of heart failure burden for the general population. Epidemiological studies of heart failure vary in study design and clinical definition, complicating efforts to produce comparable estimates of disease burden. For example, definitions of heart failure are heterogeneous and include clinical criteria established before non-invasive imaging was widely available, such as the Framingham and the European Society of Cardiology criteria. Some population-based studies also identify heart failure by International Classification of Disease (ICD) or Read codes, which have been shown to vary in some populations from classic clinical criteria level,^12^ and reveal differences between estimates of heart failure prevalence or incidence when applying different clinical scores.

Beginning in 2015, the GBD study has performed an annual systematic review of the literature from 1990 onward to identify all primary data sources with population-representative estimates of the prevalence, incidence or survival rates of heart failure. For this current analysis, we searched PubMed using structured search criteria from 1990 to 2020. Additionally, we included papers sent to us via the network of over 3500 GBD study collaborators or identified in the citations of high-impact studies identified by expert reviewers.

To ensure comparability between data sources, the GBD study defines a gold-standard case definition for each of its 370 reported causes. The case definition for heart failure was that of a clinical diagnosis of heart failure using structured criteria such as the Framingham, European Society of Cardiology or Boston criteria. Heart failure identified by ICD, International Classification of Primary Care (ICPC) or Read codes was included if the diagnosis was verified by a physician. This definition captures the American College of Cardiology/American Heart Association stage C and D, which includes patients with prior or current heart failure, regardless of treatment status.

We screened the titles and abstract of all studies for relevance, the presence of data of interest and study type. In full-text review, we screened for representativeness, diagnostic criteria and epidemiological methodology. We excluded papers that focused only on subpopulations like veterans, data that were not representative and biased geographic selections. Sampled study groups were included as long as sampling resulted in a representative population. We additionally excluded papers without extractable data, such as descriptive reports of registries or heart failure patients, or data at the wrong demographic level, such as estimates of heart failure prevalence stratified by ejection fraction.

We extracted estimates of prevalence, incidence and mortality, defined as case-fatality, with-condition mortality rate, excess mortality rate or standardised mortality ratios. We report first author, publication date, data measure, diagnosis used to identify heart failure, case ascertainment strategy and any demographic restrictions. Additionally, we report estimates of prevalence, incidence and 1 year case fatality, collapsed into the broadest available age and sex categories. When estimates were only available in detailed age or sex categories (such as 10-year age groups or both sexes), we calculated effective sample sizes from reported SE based on the Wilson Score Interval, and then collapsed cases and sample sizes to re-estimate a mean value. Site-years were calculated as the sum of years covered by study, measure and location (eg, Cuthbert *et al*, 2019, contributes three site-years to the UK as it reports data between 2015 and 2017).

Title/Abstract screening and full-text extraction were performed by separate reviewers. All included papers were reviewed by CJ and GR. We present the full list of studies evaluated in the systematic review in the online supplemental material. Neither patients nor the general public were involved in the design or conduct of this systematic review of the literature.

10.1136/heartjnl-2021-320131.supp1 Supplementary data

## Results

The PubMed search returned 42 360 studies through 15 May 2020, of which 790 were selected for full-text review and 125 included (online supplemental figure 1). Forty-five sources reported estimates of prevalence, 41 reported estimates of incidence and 58 reported estimates of mortality (tables 1–3). The included studies were published between 1991 and 2019 and represent 51 countries and 911 site-source-years of data.

**Figure 1:**
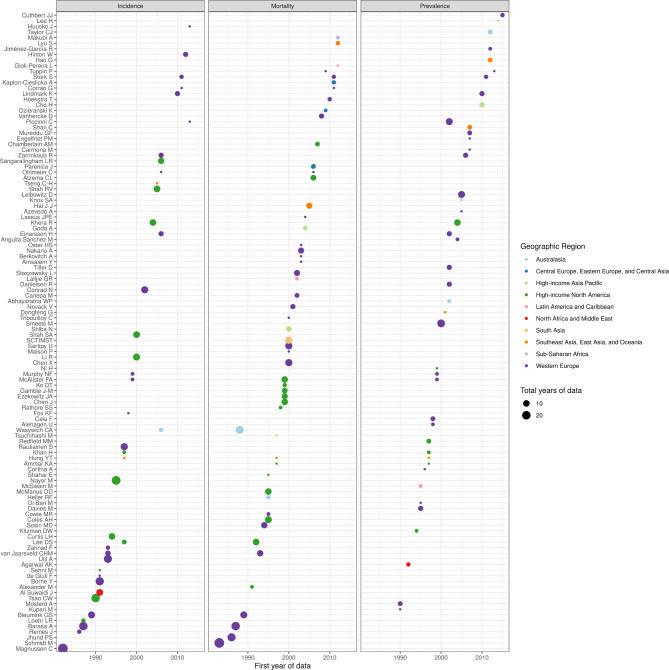
Reported prevalence of heart failure in 45 studies identified in systematic review.

**Table 1:** Studies reporting heart failure prevalence identified in systematic review

	Study	Location	Diagnostic criteria	Setting	Age range
**High-income**	Cuthbert, 2019	East Yorkshire, UK	Read codes for signs and symptoms	Patients from a single practice	
	Leibowitz, 2019	Israel	Signs and symptoms	Cohort from Jerusalem Longitudinal Cohort Study	Born 1920–1921
	Lindmark, 2019	Sweden	ICD-10 codes	Electronic medical records	18+
	Smeets, 2019	Belgium	ICPC codes	Patients in participating hospitals	
	Cho, 2018	Republic of Korea	ICD-10 codes	Health insurance patient sample (HIRA-NPS)	19+
	Danielsen, 2017	Reykjavik, Iceland	AGES-Reykjavikstudy criteria	Random sample from census	Born 1907–1935
	Einarsson, 2017	Iceland	Ageing Study criteria	Hjartavern’s Ageing Study	
	Khera, 2017	USA	ICD-9 codes	Representative sample of Medicare records	65+
	Piccinni, 2017	Italy	ICD-9 codes	Patients from participating hospitals	14+
	Stork, 2017	Germany	ICD-10 codes	Insurance records	
	Taylor, 2017	Australia	ICPC codes	Patients from randomly sampled practices	45+
	Lee, 2016	Republic of Korea	ICD-10 codes	Health insurance records	19+
	Tuppin, 2016	France	ICD-10 codes	Insurance records	
	Jiménez-García, 2014	Madrid, Spain	Chart extraction	Public health system database	
	Khan, 2014	USA	Signs and symptoms	Random sample of Medicare beneficiaries	65+
	Tiller, 2013	Germany	Signs and symptoms	Cohort study in one community	
	Zarrinkoub, 2013	Stockholm, Sweden	ICD-10 codes	Public health system database	
	Mureddu, 2012	Lazio, Italy	ESC 2005 criteria	Random sample by mail	65–84
	Carmona, 2011	Madrid, Spain	ICPC codes	Electronic medical records	14+
	Engelfriet, 2011	The Netherlands	ICPC codes and E-codes	Representative general practice registries	
	Leibowitz, 2011	Israel	Signs and symptoms	Cohort from Jerusalem Longitudinal Cohort Study	Born 1920–1921
	Alehagen, 2009	Southeast Sweden	Signs and symptoms	Survey of rural municipality	70–80
	Anguita Sanchez, 2008	Spain	Framingham criteria	Registry of participating hospitals	45+
	Knox, 2008	Australia	Signs and symptoms	Patients in randomly sampled practices	
	Ammar, 2007	Minnesota, USA	Framingham criteria	Random sample of county	45+
	Abhayaratna, 2006	Canberra, Australia	Self-report verified by record review	Random sample from electoral roll	60–85
	Azevedo, 2006	Porto, Portugal	Signs and symptoms	Population health survey	45+
	Ceia, 2005	Portugal	ESC 1995 criteria	Random sampling, primary care centres	25+
	Di Bari, 2004	Dicomano, Italy	ESC 1995 criteria	Survey of the elderly in small town	65+
	McAlister, 2004	Scotland	Read codes for signs and symptoms	Patients from participating hospitals	
	Murphy, 2004	Scotland	Read codes for signs and symptoms	Patients from participating hospitals	18+
	Ni, 2003	USA	Self-report	National health statistics	18+
	Redfield, 2003	Minnesota, USA	Chart extraction	Random sample of single county	45+
	Ceia, 2002	Madeira, Portugal	ESC 1995 criteria	Random sampling, primary care centres	25+
	Cortina, 2001	Asturias, Spain	Signs and symptoms	Random sample from census	40+
	Davies, 2001	West Midlands, England	ESC 1995 criteria	Sample from primary health centres	45+
	Kitzman, 2001	USA	Signs and symptoms	Recruitment from participating field centres	65+
	Mosterd, 1999	Rotterdam, The Netherlands	Signs and symptoms	Cohort study of single suburb	55+
	Kupari, 1997	Helsinki, Finland	Signs and symptoms	Random sampling of residents	Born 1904, 1909 or 1914
	Kannel, 1991	USA	Framingham criteria	Framingham study	
**Latin America and Caribbean**	McSwain, 1999	Antigua and Barbuda	ICD-10 codes	Patients from referral hospital	
**North Africa and Middle East**	Agarwal, 2001	Dhakliya, Oman	Signs and symptoms	Patients in referral hospital	13+
**Southeast Asia, East Asia and Oceania**	Hao, 2019	China	Modified ESC 2016 including self-report	Random sample	15+
	Shan, 2014	China	Signs and symptoms	Random sampling	60+
	Dongfeng, 2003	China	Self-report	Random sample	35–74
	Hung, 2000	China	ICD-9 codes	Patients in 11 participating hospitals	

AGES, age, gene/environment susceptibility; ESC, European Society of Cardiology; HIRA-NPS, Health Insurance Review and Assessment Service-National Patient Samples; ICD, International Classification of Disease.

**Table 2:** Studies reporting heart failure incidence identified in systematic review

	Study	Location	Diagnostic criteria	Setting	Age range
**Central Europe, Eastern Europe and Central Asia**	Rywik, 1999	Poland	ICD-9 codes	National healthcare records	
**High-income**	Huusko, 2019	Southwest Finland	ICD-10 codes	Electronic medical records	18+
	Li, 2019	USA	Signs and symptoms	Sample from existing population-based studies	40+
	Lindmark, 2019	Sweden	ICD-10 codes	Electronic medical records	18+
	Magnussen, 2019	Western Europe	Self-report, signs and symptoms or ICD-10	Patients in four cohort studies	
	Uijl, 2019	The Netherlands	ICD-9 and ICD-10 codes	Two cohort studies (MORGEN, Prospect)	
	Conrad, 2018	UK	ICD-10 codes	Electronic medical records	16+
	Hinton, 2018	England	Read codes for signs and symptoms	Patients in 164 participating centres	18+
	Shah, 2018	Massachusetts, USA	Framingham criteria	Framingham offspring study	
	Tsao, 2018	USA	Framingham criteria	Framingham original and offspring study	
	Einarsson, 2017	Iceland	Ageing Study criteria	Hjartavern’s Ageing Study	
	Khera, 2017	USA	ICD-9 codes	Representative sample of Medicare records	65+
	Piccinni, 2017	Italy	ICD-9 codes	Patients from participating hospitals	14+
	Stork, 2017	Germany	ICD-10 codes	Insurance records	
	Nayor, 2016	Massachusetts, USA	Framingham criteria	Framingham offspring study	
	Sangaralingham, 2016	USA	ICD-9 codes	Commercial insurance database	
	Ohlmeier, 2015	Germany	ICD-10 codes	Insurance records	
	Barasa, 2014	Sweden	ICD-9 and ICD-10 codes	Hospital discharges, death registry	18–84
	Borne, 2014	Malmo, Sweden	ICD-9 and ICD-10 codes	Cohort study (MDC)	Born 1923–1950
	Corrao, 2014	Lombardy, Italy	ICD-9 codes	Health services database	
	Khan, 2014	USA	Signs and symptoms	Random sample of Medicare beneficiaries	65+
	Rautiainen, 2013	Sweden	ICD-10 codes	Cohort study of two counties	Women Born 1914–1948
	Shah, 2013	USA	Cardiovascular Health Study criteria	Cohort study in six communities (MESA)	
	Zarrinkoub, 2013	Stockholm, Sweden	ICD-10 codes	Public health system database	
	Wasywich, 2010	New Zealand	ICD-9 codes	Public health system database	18+
	Curtis, 2008	USA	ICD-9 codes	Representative sample of Medicare records	65+
	Loehr, 2008	USA	ICD-9 codes	Population-based cohort (ARIC)	45–64
	van Jaarsveld, 2006	Northern Netherlands	ICPC codes	Sample from participating GPs	57+
	de Giuli, 2005	UK	Chart extraction	Sample from general practice database	45+
	Bleumink, 2004	Rotterdam, The Netherlands	European Society of Cardiology 2001 criteria	Cohort study of single suburb	55+
	Lee, 2004	Canada	ICD-9 codes	Hospital discharges, death registry	20–105
	McAlister, 2004	Scotland	Read codes for signs and symptoms	Patients from participating hospitals	
	Murphy, 2004	Scotland	Read codes for signs and symptoms	Patients from participating hospitals	18+
	Fox, 2001	South London, UK	European Society of Cardiology 1995 criteria	Registry of participating practices	
	Senni, 1999	Minnesota, USA	Framingham criteria	Random sample of single county	
	Zannad, 1999	Lorraine, France	Signs and symptoms	Patients from participating hospitals	20–80
	Remes, 1992	Eastern Finland	Boston criteria	Patients from participating hospitals	45–74
	Kannel, 1991	USA	Framingham criteria	Framingham study	
**North Africa and Middle East**	Al Suwaidi, 2004	Qatar	Framingham criteria	Patients in referral hospital	
**Southeast Asia, East Asia and Oceania**	Tseng, 2011	Taiwan (Province of China)	ICD-9 codes	Random sample of insurance registrar	20+
	Hung, 2000	China	ICD-9 codes	Patients in 11 participating hospitals	

ARIC, Atherosclerosis Risk in Communities Study; GP, general practitioner; ICD, International Classification of Disease; MDC, Malmö Diet and Cancer; MESA, Multi-Ethnic Study of Atherosclerosis; MORGEN, Monitoring Project on Risk Factors for Chronic Diseases.

**Table 3:** Studies reporting heart failure mortality identified in systematic review

	Study	Location	Diagnostic criteria	Setting	Age range
**Central Europe, Eastern Europe and Central Asia**	Kaplon-Cieslicka, 2016	Poland	ESC 2012 criteria	Polish cohort of ESC registry	18+
	Ozieranski, 2016	Poland	ESC 2012 criteria	Polish cohort of ESC registry	18+
	Parenica, 2013	Czechia	Signs and symptoms	Patients from participating hospitals	
**High-income**	Canepa, 2019	Italy	Signs and symptoms	Randomised nested trial (GISSI-HF)	
	Chen, 2019	Sweden	ICD-10 codes	Swedish Heart Failure Registry	
	Stork, 2017	Germany	ICD-10 codes	Insurance records	
	Nakano, 2016	Denmark	ICD-10 codes	National healthcare records (DHFR)	18+
	Schmidt, 2016	Denmark	ICD-8 and ICD-10 codes	Hospital discharges, death registry	
	Staszewsky, 2016	Lombardy, Italy	Chart extraction	Linked healthcare databases	
	Atzema, 2015	Canada	ICD-10 codes	Citizen registrar, healthcare databases	
	Berkovitch, 2015	Israel	Signs and symptoms	Survey of patients in 25 hospitals	
	Coles, 2015	Massachusetts, USA	Framingham criteria	Patients in 11 contributing centres	18+
	Ohlmeier, 2015	Germany	ICD-10 codes	Insurance records	
	Vanhercke, 2015	Belgium	ESC 2015 criteria	Patients in participating hospitals	18+
	Barasa, 2014	Sweden	ICD-9 and ICD-10 codes	Hospital discharges, death registry	18–84
	Corrao, 2014	Lombardy, Italy	ICD-9 codes	Health services database	
	Sartipy, 2014	Sweden	ICD-10 codes	Swedish Heart Failure Registry	
	Tuppin, 2014	France	ICD-10 codes	Insurance records	
	Chamberlain, 2013	Minnesota, USA	Framingham criteria	Electronic medical records	
	Hoekstra, 2013	The Netherlands	ESC 2008 criteria	Patients in 17 participating hospitals	18+
	Lassus, 2013	Finland	Signs and symptoms	Patients from participating hospitals	
	Maison, 2013	France	WHO classification	Patients from participating hospitals	
	McAlister, 2013	Alberta, Canada	ICD-9 and ICD-10 codes	Linked healthcare registries	18+
	McManus, 2013	Massachusetts, USA	Framingham criteria	Patients from participating hospitals	18+
	Nakano, 2013	Denmark	ICD-10 codes	National healthcare records (DHFR)	18+
	Oster, 2013	Israel	Signs and symptoms	Patients from participating hospitals	
	Chen, 2011	USA	ICD-9 codes	Review of Medicare claims data (CMS)	65+
	Ezekowitz, 2011	Alberta, Canada	ICD-9 and ICD-10 codes	Linked healthcare registries	
	Gamble, 2011	Alberta, Canada	ICD-9 and ICD-10 codes	Linked healthcare registries	
	Goda, 2010	Tokyo, Japan	Signs and symptoms	Patients in referral hospital	
	Novack, 2010	Israel	ICD-9 codes	Patients from participating hospitals	
	Tribouilloy, 2010	France	Framingham and ESC 1995 criteria	Patients from participating hospitals	20+
	Wasywich, 2010	New Zealand	ICD-9 codes	Public health system database	18+
	Jhund, 2009	Scotland	ICD-9 and ICD-10 codes	Hospital discharges, death registry	
	Amsalem, 2008	Israel	Signs and symptoms	Heart Failure Survey in Israel database	
	Ko, 2008	Ontario, Canada	Framingham criteria	Records from participating hospitals	20–105
	Shiba, 2008	Japan	Signs and symptoms	Patients in participating hospitals (CHART-1)	18+
	Ammar, 2007	Minnesota, USA	Framingham criteria	Random sample of county	45+
	Rathore, 2006	USA	ICD-9 codes	Sample of Medicare beneficiaries	65+
	van Jaarsveld, 2006	Northern Netherlands	ICPC codes	Sample from participating GPs	57+
	Bleumink, 2004	Rotterdam, The Netherlands	ESC 2001 criteria	Cohort study of single suburb	55+
	Lee, 2004	Ontario, Canada	ICD-9 codes	Hospital discharges, death registry	65+
	Shahar, 2004	Minnesota, USA	ICD-9 codes	Patients in participating counties	35–84
	Sosin, 2004	UK	ESC 2001 criteria	Patients in participating hospitals	
	Lee, 2003	Ontario, Canada	ICD-9 codes	Patients from participating hospitals	
	Cowie, 2000	West London, England	Adapted ESC 1995 criteria	Patients in district hospital	
	Heller, 2000	South Wales, Australia	ICD-9 and ICD-10 codes	Patients in 22 hospitals (Heart and Stroke Register)	
	Tsuchihashi, 2000	Japan	Framingham criteria	Patients from participating hospitals	
	Alexander, 1999	California, USA	ICD-9 codes	Review of CA hospital discharges	
**Latin America and Caribbean**	Gioli-Pereira, 2019	Sao Paulo, Brazil	Signs and symptoms	Patients from a single practice	18–80
	Lalljie, 2007	Jamaica	Framingham criteria	Patients from a single practice	
	AHRI, 2013	India	ESC 2012 criteria	Patients from participating hospitals	
**South Asia**	SCTIMST, 2006	India	ESC 1995 criteria	Patients from participating hospitals	
	SCTIMST, 2001	India	ESC 1995 criteria	Patients from participating hospitals	
**Southeast Asia, East Asia and Oceania**	Lyu, 2019	China	Boston criteria	Patients from participating hospitals	18+
	Hai, 2016	Hong Kong Special Administrative Region of China	Framingham criteria	Patients from a single hospital	18+
	Hung, 2000	China	ICD-9 codes	Patients in 11 participating hospitals	
**Sub-Saharan Africa**	Makubi, 2016	United Republic of Tanzania	Framingham criteria	Patients from referral hospital	18+

CHART, Chronic Heart Failure Analysis and Registry in the Tohoku District; CMS, Centers for Medicare & Medicaid Services; DHFR, Danish Heart Failure Registry; ESC, European Society of Cardiology; GISSI-HF, Gruppo Italiano per lo Studio della Streptochinasi nell'Infarto Miocardico-Heart Failure; GP, general practitioner.

Design of these studies varied. Seventeen used random sampling or surveys of entire municipalities. Thirty-nine studies used large administrative databases, such as insurance records or state-wide hospital discharges, to identify the study population. Sixteen studies were cohort-based, including the Framingham, Atherosclerosis Risk in Communities Study and Jerusalem Longitudinal Cohort study. Forty-five studies reported patients presenting to participating hospitals, such as a single referral centre or several cooperating sites.

One hundred one of the 125 included studies reported data from high-income regions, which includes Western Europe, North America, Australasia, Southern Latin America and high-income Asia Pacific. Four studies reported data from Central Europe, Eastern Europe and Central Asia; three from Latin America and the Caribbean; two from North Africa and the Middle East; three from South Asia; seven from Southeast Asia, East Asia and Oceania and one from sub-Saharan Africa. The most common locations represented were the USA (23 studies), the UK (8), China (7) and Israel (6). The demographic profile of included patients varied by study (tables 1–3). Some studies restricted to certain age groups, such as patients aged 65+ years or those born in 1920–1921, while others included patients of all ages. One study surveyed only women.

Figure 1 shows reported values of heart failure prevalence, separated by demographic profile (studies including patients of all ages; all adults, referring to patients aged 18 years and older and older adults, referring to patients 50 and older). When collapsed into the broadest reported age and sex groups, estimates of heart failure prevalence ranged from 0.002 per capita, in a Hong Kong study that enrolled hospitalised heart failure patients and estimated prevalence from the site’s catchment area, to 0.18 per capita, in a US study of Medicare beneficiaries aged 65+ years that captured heart failure with ICD codes (figure 1). The five highest prevalence values reported were from studies focusing on patients aged 50+ years. Among studies limited to older adults, the average of reported prevalence values was 8.3%. Among studies limited to all adults, average reported prevalence was 3.4%. Among studies enrolling patients of all ages, average reported prevalence was 1.3%.

The most common locations reporting prevalence were the USA (five studies), Spain (four studies), Australia (three studies), Portugal (three studies), China (three studies) and Sweden (three studies). In prevalence studies, heart failure was diagnosed by signs and symptoms (including Framingham, ESC and Boston criteria, and chart review) in 26 studies, and by ICD, ICPC or Read codes in 17 studies (table 1). Sampling techniques for these studies included random sampling from primary care centres, random sampling from official census records, review of electronic medical records and medical surveys administrated to entire towns or populations.

**Figure 2:**
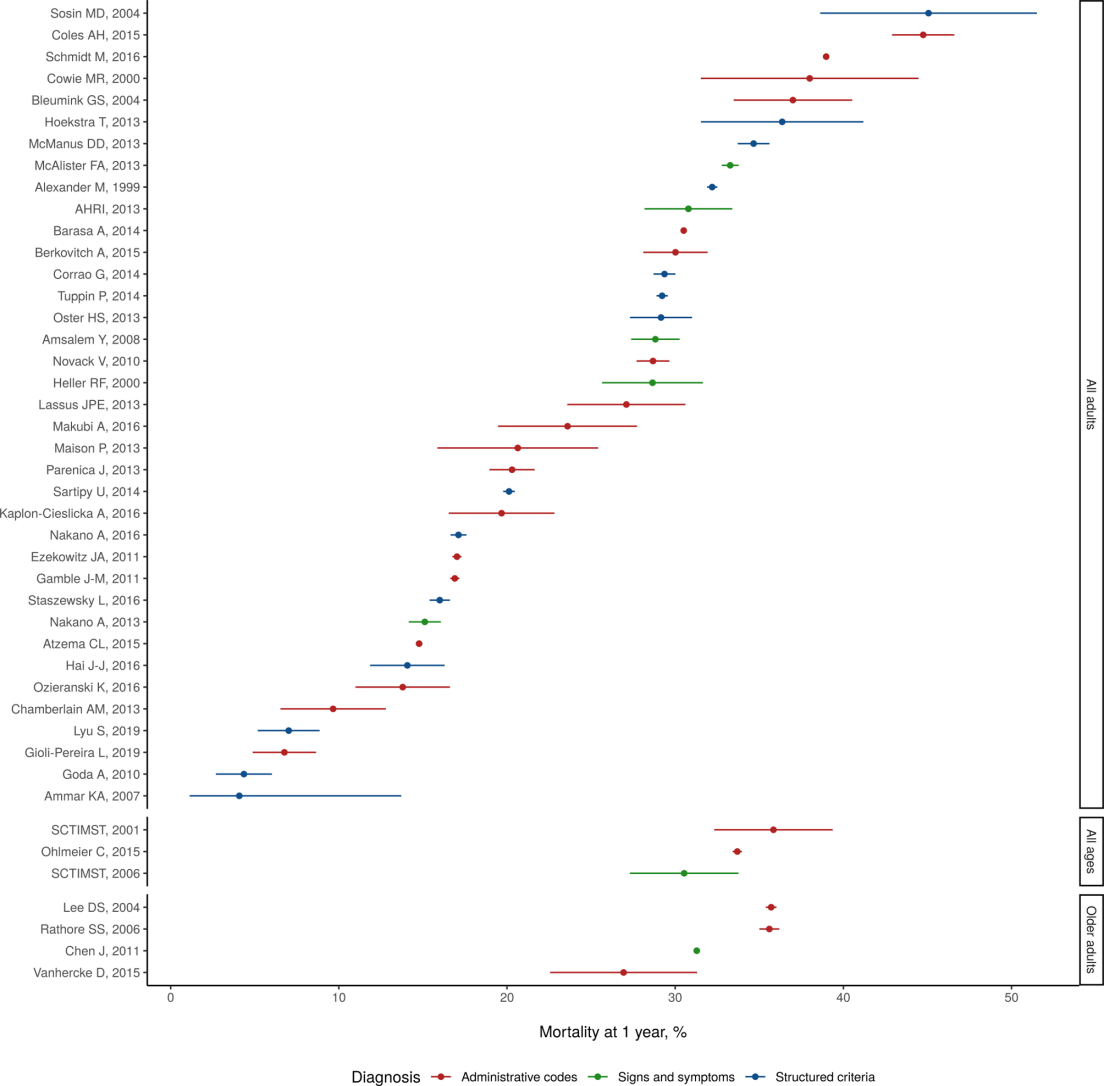
Reported incidence of heart failure in 41 studies identified in systematic review.

Figure 2 shows reported values of heart failure incidence, separated by demographic profile. Reported estimates of heart failure incidence ranged from 100/100 000 person-years, in the French EPICAL study of acute heart failure in those aged 20–80 years, to 4300/100 000 person-years in a US study of Medicare beneficiaries 65+ identifying heart failure with ICD codes (figure 2). Among studies limited to older adults, the average of reported incidence values was 1600/100 000 person-years. Among studies limited to all adults, average incidence was 840/100 000 person-years. Among studies enrolling patients of all ages, average reported incidence was 460/100 000 person-years.

Common locations reporting heart failure incidence were the USA (12 studies), the UK (6), Sweden (4) and the Netherlands (3). Heart failure incidence was diagnosed by signs and symptoms (including Framingham, ESC and Boston criteria, and chart review) in 16 studies, and by ICD, ICPC or Read codes in 25 studies (table 2). In these studies, sampling techniques included random sample of insurance registrar, hospital and death registry, population-based cohort and analysis of linked public health systems databases.

**Figure 3:**
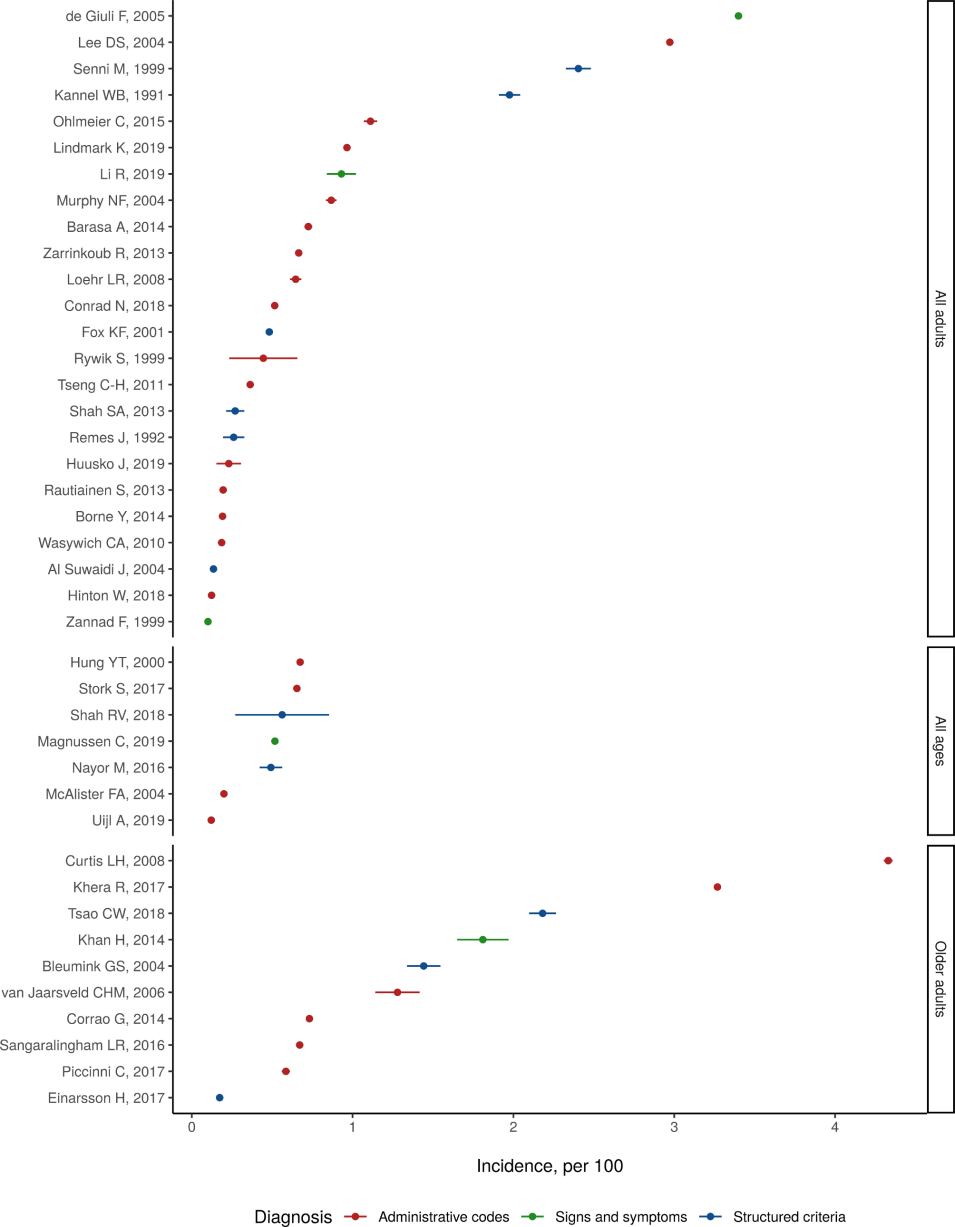
Reported 1-year case fatality of heart failure in 44 studies identified in systematic review.

Figure 3 shows reported values of 1-year heart failure case fatality, separated by demographic profile. Reported estimates of 1-year case fatality ranged from 4%, in a study that randomly sampled Minnesota residents, to 45%, in a 1994 study of acute heart failure admissions in Birmingham (figure 3). Among studies limited to older adults, the average of reported 1-year case fatality values was 33%. Among studies limited to all adults, average reported 1-year case fatality was 24%. Among studies enrolling patients of all ages, average reported 1-year case fatality was 33%.

Common locations reporting heart failure case fatality were the USA (seven studies), Canada (6), India (5) and Israel (4). One-year heart failure case fatality was diagnosed by signs and symptoms (including Framingham, ESC and Boston criteria, and chart review) in 25 studies, and by ICD, ICPC or Read codes in 23 studies (table 3). In these studies, sampling techniques included random sample of primary or specialty care centres, review of electronic medical records or insurance records and medical surveys administrated to entire towns or populations.

**Figure 4:**
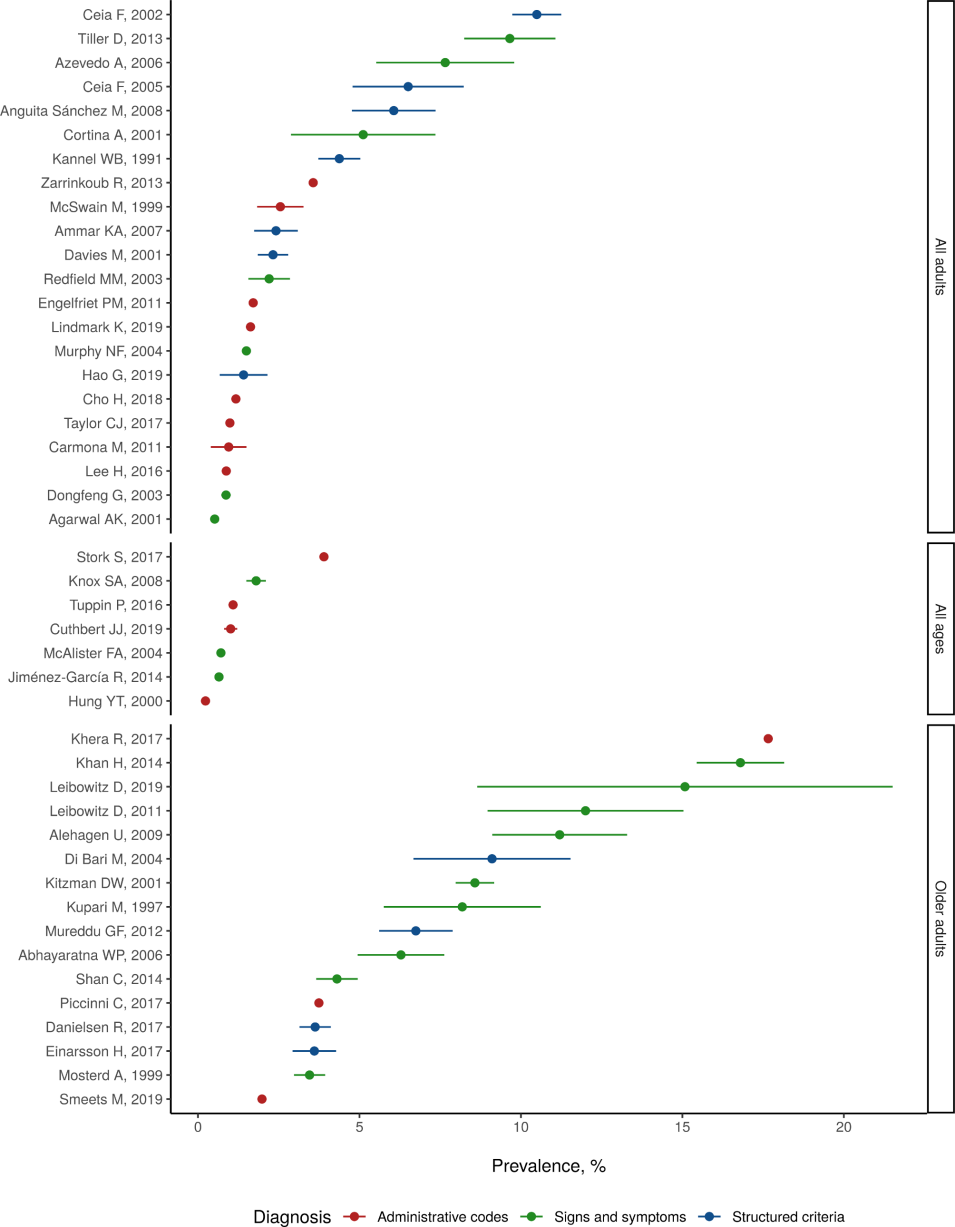
Data coverage by year, measure and geographic region.

Studies from 23 countries report estimates of heart failure prevalence or incidence (online supplemental figure 2). Additionally, studies from 23 countries report estimates of heart failure mortality (online supplemental figure 3). Figure 4 shows the number of data-years contributed by each study, coloured by geographic region. Of 911 total site-years of data, 817 were from high-income locations (figure 4). Studies varied in case ascertainment criteria, heart failure diagnosis type, epidemiological design and demographic breakdown. Several papers reported on long-running studies like Framingham or the AGES-Reykjavik study, while others were estimates from a single year or site. Many studies included all patients managed for heart failure by participating hospitals, general practitioners or clinics; these often provided an estimate of catchment area to calculate prevalence or incidence. Some studies used large insurance databases or national administrative healthcare records to identify heart failure patients. Still others were reports of community-based surveys that invited patients to conduct a health screen and heart failure assessment. Many studies did not report specific diagnostic criteria beyond physician diagnosis and are noted as ‘signs and symptoms’ in the table. The age and sex breakdown of heart failure cases and sample sizes differed by study and were not always reported in granular detail; aggregated estimates reflect this variation.

## Discussion

Our prospective systematic review identified 125 studies reporting prevalence, incidence or mortality of heart failure, synthesising the landscape of epidemiological research on heart failure. Data reported in these studies will inform the GBD 2020 study, help elucidate the global epidemiology of heart failure and guide resources, research and interventions.

These studies describe a prevalence and incidence of heart failure that varies widely across locations. Much of the observed variation may reflect true changes in the age-specific burden of heart failure within specific populations. Our results suggest that differences in study design and case ascertainment strategy may also contribute to the observed heterogeneity. Heart failure remains a condition frequently identified when patients develop acute symptoms and, at times, are clinically unstable. Especially relevant are differences in diagnostic criteria, whose sensitivity and specificities reflect clinical judgement across diverse and complex settings such as emergency departments and primary care offices. While some studies apply research-grade enrolment protocols in these settings or even extend surveillance to households, many remain simple counts of acute decompensation of heart failure. As technologies for non-invasive evaluation of heart failure improve, there is a need to shift studies of heart failure epidemiology from case identification based on physical examination and cardiac auscultation to a standardised application of rapid, inexpensive and robust laboratory and echocardiographic criteria.

While the GBD has developed methodology to estimate and correct for systematic bias between case definitions,^13 14^ alignment of standards for epidemiological studies of heart failure would improve the comparability between studies and reduce the need for statistical bias correction. National and international societies could help align criteria for epidemiological purposes similar to standardised reporting used for cardiac arrest and myocardial infarction, and standard data collection methods could be adopted for health surveys for non-communicable diseases. Additionally, this review presents collapsed estimates, not ones standardised to a reference population, so heterogeneity in population structures remain present in the summarised estimates.

High-quality data from more geographical regions is also necessary to understand global patterns and the manner in which diverse pathophysiological aetiologies may affect patterns of heart failure. Although this review identified data from 51 countries, only 11 countries were outside of the high-income world: the Czech Republic, Poland, Antigua and Barbuda, Jamaica, Brazil, Oman, Qatar, India, China, Taiwan and Tanzania. Together, only 94 of 911 site-years of data were outside of the high-income world. Given this, covariates and statistical models are necessary to make estimates of the burden of heart failure in countries or regions where there is limited data. Further investments in data collection and population-based surveys in such locations would improve our understanding of global patterns. Additional data are also needed to better understand the causal pathways by which a wide variety of cardiovascular and other diseases drive the incidence of heart failure, and how these conditions vary across regions in their overall contribution to heart failure prevalence.

## Conclusion

Prevalence, incidence and survival for heart failure varied widely across countries and studies, reflecting a range of study design. Heart failure remains a high prevalence disease among older adults with a high risk of death at 1 year. This review synthesises all available published estimates of heart failure burden. Future efforts will include the use of geospatial statistical models to produce estimates of global disease burden due to heart failure. Given its place as a common final pathway for a broad set of conditions, an improved understanding of heart failure in the general population would be useful to guide research, resource allocation and policy, and to inform larger efforts to reduce the burden of non-communicable diseases.
