# Supplementary material for: Prevalence, incidence and survival of heart failure: a systematic review
Source: Heart. 2022 Jan 18;108(17):1351–60. doi: 10.1136/heartjnl-2021-320131 (PMC9380485; doi:10.1136/heartjnl-2021-320131)
Supplement: Supplementary data [file heartjnl-2021-320131supp001.pdf]

Supplemental Material

In this supplement, we present the full list of studies evaluated in the systematic review and supplemental tables and figures.

References

Abhayaratna WP, Becker NG, Smith WT, Marwick TH, Jeffery IM, McGill DA. Prevalence of heart failure and systolic ventricular dysfunction in older Australians: the Canberra Heart Study. *Med J Aust* 2006; **184**: 151–4.

Agarwal AK, Venugopalan P, de Bono D. Prevalence and aetiology of heart failure in an Arab population. *Eur J Heart Fail* 2001; **3**: 301–5.

Al Suwaidi J, Bener A, Hajar HA, Numan MT. Does hospitalization for congestive heart failure occur more frequently in Ramadan: a population-based study (1991–2001). *Int J Cardiol* 2004; **96**: 217–21.

Alehagen U, Ericsson A, Dahlström U. Are There Any Significant Differences Between Females and Males in the Management of Heart Failure? Gender Aspects of an Elderly Population With Symptoms Associated With Heart Failure. *J Card Fail* 2009; **15**: 501–7.

Alexander M, Grumbach K, Remy L, Rowell R, Massie BM. Congestive heart failure hospitalizations and survival in California: Patterns according to race/ethnicity. *Am Heart J* 1999; **137**: 919–27.

Ammar KA, Jacobsen SJ, Mahoney DW, *et al.* Prevalence and Prognostic Significance of Heart Failure Stages: Application of the American College of Cardiology/American Heart Association Heart Failure Staging Criteria in the Community. *Circulation* 2007; **115**: 1563–70.

Amsalem Y, Garty M, Schwartz R, *et al.* Prevalence and significance of unrecognized renal insufficiency in patients with heart failure. *Eur Heart J* 2008; **29**: 1029–36.

Ananthapuri Hospitals and Research Institute (AHRI) (India), Cosmopolitan Hospital (India), Government Medical College, Thiruvananthapuram, Jubilee Memorial Hospital (India). India - Trivandrum Heart Failure Registry 2013. .

Anguita Sánchez M, Crespo Leiro MG, de Teresa Galván E, Jiménez Navarro M, Alonso-Pulpón L, Muñiz García J. Prevalence of Heart Failure in the Spanish General Population Aged Over 45 Years. The PRICE Study. *Rev Esp Cardiol Engl Ed* 2008; **61**: 1041–9.

Atzema CL, Khan S, Lu H, *et al.* Cardiovascular Disease Rates, Outcomes, and Quality of Care in Ontario Métis: A Population-Based Cohort Study. *PLOS ONE* 2015; **10**: e0121779.

Azevedo A. Population based study on the prevalence of the stages of heart failure. *Heart* 2006; **92**: 1161–3.

Barasa A, Schaufelberger M, Lappas G, Swedberg K, Dellborg M, Rosengren A. Heart failure in young adults: 20-year trends in hospitalization, aetiology, and case fatality in Sweden. *Eur Heart J* 2014; **35**: 25–32.

Berkovitch A, Maor E, Sabbag A, *et al.* Precipitating Factors for Acute Heart Failure Hospitalization and Long-Term Survival. *Medicine (Baltimore)* 2015; **94**: e2330.

Bleumink GS, Knetsch AM, Sturkenboom MCJM, *et al.* Quantifying the heart failure epidemic: prevalence, incidence rate, lifetime risk and prognosis of heart failure The Rotterdam Study. *Eur Heart J* 2004; **25**: 1614–9.

Borné Y, Hedblad B, Essén B, Engström G. Anthropometric measures in relation to risk of heart failure hospitalization: a Swedish population-based cohort study. *Eur J Public Health* 2014; **24**: 215–20.

Canepa M, Ameri P, Lucci D, *et al.* Modes of death and prognostic outliers in chronic heart failure. *Am Heart J* 2019; **208**: 100–9.

Carmona M, Garcia-Olmos LM, Alberquilla A, *et al.* Heart failure in the family practice: a study of the prevalence and co-morbidity. *Fam Pract* 2011; **28**: 128–33.

Ceia F, Fonseca C, Azevedo I, *et al.* Epidemiologia da Insuficiência Cardíaca em Cuidados Primários na Região Autónoma da Madeira: o Estudo EPICA-RAM [11]. *Rev Port Cardiol* 2005; **24**: 17.

Ceia F, Fonseca C, Mota T, *et al.* Prevalence of chronic heart failure in Southwestern Europe: the EPICA study. *Eur J Heart Fail* 2002; **4**: 531–9.

Chamberlain AM, McNallan SM, Dunlay SM, *et al.* Physical Health Status Measures Predict All-Cause Mortality in Patients With Heart Failure. *Circ Heart Fail* 2013; **6**: 669–75.

Chen J, Normand S-LT, Wang Y, Krumholz HM. National and Regional Trends in Heart Failure Hospitalization and Mortality Rates for Medicare Beneficiaries, 1998-2008. *JAMA* 2011; **306**: 1669.

Chen X, Savarese G, Dahlström U, Lund LH, Fu M. Age-dependent differences in clinical phenotype and prognosis in heart failure with mid-range ejection compared with heart failure with reduced or preserved ejection fraction. *Clin Res Cardiol* 2019; **108**: 1394–405.

Cho H, Oh S-H, Lee H, Cho H-J, Kang H-Y. The incremental economic burden of heart failure: A population-based investigation from South Korea. *PLOS ONE* 2018; **13**: e0208731.

Coles AH, Tisminetzky M, Yarzebski J, *et al.* Magnitude of and Prognostic Factors Associated With 1-Year Mortality After Hospital Discharge for Acute Decompensated Heart Failure Based on Ejection Fraction Findings. *J Am Heart Assoc* 2015; **4**. DOI:10.1161/JAHA.115.002303.

Conrad N, Judge A, Tran J, *et al.* Temporal trends and patterns in heart failure incidence: a population-based study of 4 million individuals. *The Lancet* 2018; **391**: 572–80.

Corrao G, Ghirardi A, Ibrahim B, Merlino L, Maggioni AP. Burden of new hospitalization for heart failure: a population-based investigation from Italy: Burden of new hospitalization for heart failure. *Eur J Heart Fail* 2014; **16**: 729–36.

Corrao G, Ghirardi A, Ibrahim B, Merlino L, Maggioni AP. Burden of new hospitalization for heart failure: a population-based investigation from Italy: Burden of new hospitalization for heart failure. *Eur J Heart Fail* 2014; **16**: 729–36.

Cortina A, Reguero J, Segovia E, *et al.* Prevalence of heart failure in Asturias (a region in the North of Spain). *Am J Cardiol* 2001; **87**: 1417–9.

Cowie MR. Survival of patients with a new diagnosis of heart failure: a population based study. *Heart* 2000; **83**: 505–10.

Curtis LH. Incidence and Prevalence of Heart Failure in Elderly Persons, 1994-2003. *Arch Intern Med* 2008; **168**: 418.

Cuthbert JJ, Gopal J, Crundall-Goode A, Clark AL. Are there patients missing from community heart failure registers? An audit of clinical practice. *Eur J Prev Cardiol* 2019; **26**: 291–8.

Danielsen R, Thorgeirsson G, Einarsson H, *et al.* Prevalence of heart failure in the elderly and future projections: the AGES-Reykjavík study. *Scand Cardiovasc J* 2017; **51**: 183–9.

Davies M, Hobbs F, Davis R, *et al.* Prevalence of left-ventricular systolic dysfunction and heart failure in the Echocardiographic Heart of England Screening study: a population based study. *The Lancet* 2001; **358**: 439–44.

de Giuli F, Khaw K-T, Cowie MR, Sutton GC, Ferrari R, Poole-Wilson PA. Incidence and outcome of persons with a clinical diagnosis of heart failure in a general practice population of 696,884 in the United Kingdom. *Eur J Heart Fail* 2005; **7**: 295–302.

Di Bari M, Pozzi C, Cavallini MC, *et al.* The diagnosis of heart failure in the community. *J Am Coll Cardiol* 2004; **44**: 1601–8.

Einarsson H, Thorgeirsson G, Danielsen R, Olafsson O, Aspelund T, Gudnason V. [Heart failure among elderly Icelanders: Incidence, prevalence, underlying diseases and long-term survival]. *Laeknabladid* 2017; **103**: 429–36.

Engelfriet PM, Hoogenveen RT, Boshuizen HC, van Baal PHM. To die with or from heart failure: a difference that counts: Is heart failure underrepresented in national mortality statistics? *Eur J Heart Fail* 2011; **13**: 377–83.

Ezekowitz JA, Kaul P, Bakal JA, Quan H, McAlister FA. Trends in heart failure care: has the incident diagnosis of heart failure shifted from the hospital to the emergency department and outpatient clinics? *Eur J Heart Fail* 2011; **13**: 142–7.

Fox K. Coronary artery disease as the cause of incident heart failure in the population. *Eur Heart J* 2001; **22**: 228–36.

Gamble J-M, Eurich DT, Ezekowitz JA, Kaul P, Quan H, McAlister FA. Patterns of Care and Outcomes Differ for Urban Versus Rural Patients With Newly Diagnosed Heart Failure, Even in a Universal Healthcare System. *Circ Heart Fail* 2011; **4**: 317–23.

Gioli-Pereira L, Marcondes-Braga FG, Bernardez-Pereira S, *et al.* Predictors of one-year outcomes in chronic heart failure: the portrait of a middle income country. *BMC Cardiovasc Disord* 2019; **19**: 251.

Goda A, Yamashita T, Suzuki S, *et al.* Heart failure with preserved versus reduced left ventricular systolic function: A prospective cohort of Shinken Database 2004–2005. *J Cardiol* 2010; **55**: 108–16.

Gu D, Huang G, He J. Investigation of prevalence and distributing feature of chronic heart failure in Chinese adult populatio. *Zhonghua Xin Xue Guan Bing Za Zhi* 2003; **31**: 3–6.

Hai J-J, Chan P-H, Huang D, *et al.* Clinical Characteristics, Management, and Outcomes of Hospitalized Heart Failure in a Chinese Population—The Hong Kong Heart Failure Registry. *J Card Fail* 2016; **22**: 600–8.

Hao G, Wang X, Chen Z, *et al.* Prevalence of heart failure and left ventricular dysfunction in China: the China Hypertension Survey, 2012–2015. *Eur J Heart Fail* 2019; **21**: 1329–37.

Heller RF, Fisher JD, O'Este CA, Lim LL, Dobson AJ, Porter R. Death and readmission in the year after hospital admission with cardiovascular disease: the Hunter Area Heart and Stroke Register. *Med J Aust* 2000; **172**: 261–5.

Hinton W, McGovern A, Coyle R, *et al.* Incidence and prevalence of cardiovascular disease in English primary care: a cross-sectional and follow-up study of the Royal College of General Practitioners (RCGP) Research and Surveillance Centre (RSC). *BMJ Open* 2018; **8**: e020282.

Hoekstra T, Jaarsma T, van Veldhuisen DJ, Hillege HL, Sanderman R, Lesman-Leegte I. Quality of life and survival in patients with heart failure. *Eur J Heart Fail* 2013; **15**: 94–102.

Hung Y, Cheung N, Ip S, Fung H. Epidemiology of heart failure in Hong Kong, 1997. ; : 4.

Huusko J, Kurki S, Toppila I, *et al.* Heart failure in Finland: clinical characteristics, mortality, and healthcare resource use. *ESC Heart Fail* 2019; **6**: 603–12.

Jhund PS, MacIntyre K, Simpson CR, *et al.* Long-Term Trends in First Hospitalization for Heart Failure and Subsequent Survival Between 1986 and 2003: A Population Study of 5.1 Million People. *Circulation* 2009; **119**: 515–23.

Jiménez-García R, Esteban-Vasallo MD, Rodríguez-Rieiro C, *et al.* Coverage and predictors of vaccination against 2012/13 seasonal influenza in Madrid, Spain: Analysis of population-based computerized immunization registries and clinical records. *Hum Vaccines Immunother* 2014; **10**: 449–55.

Kannel WB, Belanger AJ. Epidemiology of heart failure. *Am Heart J* 1991; **121**: 951–7.

Kapłon-Cieślicka A, Tymińska A, Peller M, *et al.* Diagnosis, Clinical Course, and 1-Year Outcome in Patients Hospitalized for Heart Failure With Preserved Ejection Fraction (from the Polish Cohort of the European Society of Cardiology Heart Failure Long-Term Registry). *Am J Cardiol* 2016; **118**: 535–42.

Khan H, Kalogeropoulos AP, Zannad F, *et al.* Incident heart failure in relation to vascular disease: Insights from the Health, Aging, and Body Composition Study: Incident heart failure in relation to vascular disease. *Eur J Heart Fail* 2014; **16**: 526–34.

Khera R, Pandey A, Ayers CR, *et al.* Contemporary Epidemiology of Heart Failure in Fee-For-Service Medicare Beneficiaries Across Healthcare Settings. *Circ Heart Fail* 2017; **10**. DOI:10.1161/CIRCHEARTFAILURE.117.004402.

Kitzman DW, Gardin JM, Gottdiener JS, *et al.* Importance of heart failure with preserved systolic function in patients ≥65 years of age. *Am J Cardiol* 2001; **87**: 413–9.

Knox SA, Harrison CM, Britt HC, Henderson JV. Estimating prevalence of common chronic morbidities in Australia. *Med J Aust* 2008; **189**: 66–70.

Ko DT, Alter DA, Austin PC, *et al.* Life expectancy after an index hospitalization for patients with heart failure: A population-based study. *Am Heart J* 2008; **155**: 324–31.

Kupari M, Lindroos M, Iivanainen AM, Heikkilä J, Tilvis R. Congestive heart failure in old age: prevalence, mechanisms and 4-year prognosis in the Helsinki Ageing Study. *J Intern Med* 1997; **241**: 387–94.

Lalljie G, Lalljie S. Characteristics, treatment and short-term survival of patients with heart failure in a cardiology private practice in Jamaica. *West Indian Med J* 2007; **56**. DOI:10.1590/S0043-31442007000200007.

Lassus JPE, Siirilä-Waris K, Nieminen MS, *et al.* Long-term survival after hospitalization for acute heart failure — Differences in prognosis of acutely decompensated chronic and new-onset acute heart failure. *Int J Cardiol* 2013; **168**: 458–62.

Lee DS, Austin PC, Rouleau JL, Liu PP, Naimark D, Tu JV. Predicting Mortality Among Patients Hospitalized for Heart Failure. ; : 7.

Lee DS, Johansen H, Gong Y, Hall RE, Tu JV, Cox JL. Chapter 14: Regional outcomes of heart failure in Canada. ; : 9.

Lee DS, Mamdani MM, Austin PC, *et al.* Trends in heart failure outcomes and pharmacotherapy: 1992 to 2000. *Am J Med* 2004; **116**: 581–9.

Lee H, Oh S-H, Cho H, Cho H-J, Kang H-Y. Prevalence and socio-economic burden of heart failure in an aging society of South Korea. *BMC Cardiovasc Disord* 2016; **16**: 215.

Leibowitz D, Stessman-Lande I, Jacobs J, *et al.* Cardiac Structure and Function in Persons 85 Years of Age. *Am J Cardiol* 2011; **108**: 465–70.

Leibowitz D, Stessman-Lande I, Sliman H, Jacobs JM, Stessman J, Gilon D. Longitudinal changes in cardiac function in the very elderly: the Jerusalem longitudinal cohort study. ; : 6.

Li R, Zhang J, Gao Y, Li J, Yan B, Wang G. Impact of Lung Function and SDB on Incident Myocardial Infarction and Heart Failure: A Community-based Study. *Lung* 2019; **197**: 339–47.

Lindmark K, Boman K, Olofsson M, *et al.* Epidemiology of heart failure and trends in diagnostic work-up: a retrospective, population-based cohort study in Sweden. *Clin Epidemiol* 2019; **Volume 11**: 231–44.

Loehr LR, Rosamond WD, Chang PP, Folsom AR, Chambless LE. Heart Failure Incidence and Survival (from the Atherosclerosis Risk in Communities Study). *Am J Cardiol* 2008; **101**: 1016–22.

Lyu S, Yu L, Tan H, *et al.* Clinical characteristics and prognosis of heart failure with mid-range ejection fraction: insights from a multi-centre registry study in China. *BMC Cardiovasc Disord* 2019; **19**: 209.

Magnussen C, Niiranen TJ, Ojeda FM, *et al.* Sex-Specific Epidemiology of Heart Failure Risk and Mortality in Europe. *JACC Heart Fail* 2019; **7**: 204–13.

Maison P, Desamericq G, Hemery F, *et al.* Relationship between recommended chronic heart failure treatments and mortality over 8 years in real-world conditions: a pharmacoepidemiological study. *Eur J Clin Pharmacol* 2013; **69**: 901–8.

Makubi A, Hage C, Sartipy U, *et al.* Heart failure in Tanzania and Sweden: Comparative characterization and prognosis in the Tanzania Heart Failure (TaHeF) study and the Swedish Heart Failure Registry (SwedeHF). *Int J Cardiol* 2016; **220**: 750–8.

McAlister FA, Bakal JA, Kaul P, *et al.* Changes in Heart Failure Outcomes After a Province-Wide Change in Health Service Provision A Natural Experiment in Alberta, Canada. *Circ Heart Fail* 2013; **6**: 76–82.

McAlister FA, Murphy NF, Simpson CR, *et al.* Influence of socioeconomic deprivation on the primary care burden and treatment of patients with a diagnosis of heart failure in general practice in Scotland: population based study. *BMJ* 2004; **328**: 1110.

McManus DD, Saczynski JS, Lessard D, *et al.* Recent Trends in the Incidence, Treatment, and Prognosis of Patients With Heart Failure and Atrial Fibrillation (the Worcester Heart Failure Study). *Am J Cardiol* 2013; **111**: 1460–5.

McSwain M, Martin TC, Amaraswamy R. The prevalence, aetiology and treatment of congestive cardiac failure in Antigua and Barbuda. *West Indian Med J* 1999; **48**: 137–40.

Mosterd A. Prevalence of heart failure and left ventricular dysfunction in the general population; The Rotterdam Study. *Eur Heart J* 1999; **20**: 447–55.

Mureddu GF, Agabiti N, Rizzello V, *et al.* Prevalence of preclinical and clinical heart failure in the elderly. A population-based study in Central Italy. *Eur J Heart Fail* 2012; **14**: 718–29.

Murphy NF. National survey of the prevalence, incidence, primary care burden, and treatment of heart failure in Scotland. *Heart* 2004; **90**: 1129–36.

Nakano A, Egstrup K, Svendsen ML, *et al.* Age- and sex-related differences in use of guideline-recommended care and mortality among patients with incident heart failure in Denmark. *Age Ageing* 2016; **45**: 635–41.

Nakano A, Johnsen SP, Frederiksen BL, *et al.* Trends in quality of care among patients with incident heart failure in Denmark 2003–2010: a nationwide cohort study. *BMC Health Serv Res* 2013; **13**: 391.

Nayor M, Enserro DM, Vasan RS, Xanthakis V. Cardiovascular Health Status and Incidence of Heart Failure in the Framingham Offspring Study. *Circ Heart Fail* 2016; **9**. DOI:10.1161/CIRCHEARTFAILURE.115.002416.

Ni H. Prevalence of self-reported heart failure among US adults: results from the 1999 National Health Interview Survey. *Am Heart J* 2003; **146**: 121–8.

Novack V, Pencina M, Zahger D, *et al.* Routine Laboratory Results and Thirty Day and One-Year Mortality Risk Following Hospitalization with Acute Decompensated Heart Failure. *PLoS ONE* 2010; **5**: e12184.

Ohlmeier C, Mikolajczyk R, Frick J, Prütz F, Haverkamp W, Garbe E. Incidence, prevalence and 1-year all-cause mortality of heart failure in Germany: a study based on electronic healthcare data of more than six million persons. *Clin Res Cardiol* 2015; **104**: 688–96.

Oster HS, Benderly M, Hoffman M, Cohen E, Shotan A, Mittelman M. Mortality in Heart Failure with Worsening Anemia: A National Study. 2013; **15**: 5.

Ozierański K, Kapłon-Cieślicka A, Peller M, *et al.* Clinical characteristics and predictors of one-year outcome of heart failure patients with atrial fibrillation compared to heart failure patients in sinus rhythm. ~~*Atr Fibrillation*~~*Polish Heart J*; 2016~~: 11~~.

Parenica J, Spinar J, Vitovec J, *et al.* Long-term survival following acute heart failure: The Acute Heart Failure Database Main registry (AHEAD Main). *Eur J Intern Med* 2013; **24**: 151–60.

Piccinni C, Antonazzo IC, Simonetti M, *et al.* The Burden of Chronic Heart Failure in Primary Care in Italy. *High Blood Press Cardiovasc Prev* 2017; **24**: 171–8.

Piccinni C, Antonazzo IC, Simonetti M, *et al.* The Burden of Chronic Heart Failure in Primary Care in Italy. *High Blood Press Cardiovasc Prev* 2017; **24**: 171–8.

Rathore SS, Masoudi FA, Wang Y, *et al.* Socioeconomic status, treatment, and outcomes among elderly patients hospitalized with heart failure: Findings from the National Heart Failure Project. *Am Heart J* 2006; **152**: 371–8.

Rautiainen S, Levitan EB, Mittleman MA, Wolk A. Total Antioxidant Capacity of Diet and Risk of Heart Failure: A Population-based Prospective Cohort of Women. *Am J Med* 2013; **126**: 494–500.

Redfield MM, Jacobsen SJ, Burnett JC, Mahoney DW, Bailey KR, Rodeheffer RJ. Burden of systolic and diastolic ventricular dysfunction in the community appreciating the scope of the heart failure epidemic. *ACC Curr J Rev* 2003; **12**: 50–1.

Remes J, Reunanen A, Aromaa A, Pyörälä K. Incidence of heart failure in eastern Finland: a population-based surveillance study. *Eur Heart J* 1992; **13**: 588–93.

Rywik S, Broda G, Jasinski B. Heart Failure - Mortality and Hospital Morbidity in Polish Population. *Kardiol Pol* 1999; **50**: 20–34.

Sangaralingham LR, Shah ND, Yao X, Roger VL, Dunlay SM. Incidence and Early Outcomes of Heart Failure in Commercially Insured and Medicare Advantage Patients, 2006–2014. 2017; : 11.

Sartipy U, Dahlström U, Edner M, Lund LH. Predicting survival in heart failure: validation of the MAGGIC heart failure risk score in 51 043 patients from the Swedish Heart Failure Registry: Validation of the MAGGIC heart failure risk score in 51 043 patients. *Eur J Heart Fail* 2014; **16**: 173–9.

Schmidt M, Ulrichsen SP, Pedersen L, Bøtker HE, Sørensen HT. Thirty-year trends in heart failure hospitalization and mortality rates and the prognostic impact of co-morbidity: a Danish nationwide cohort study: Heart failure hospitalization, outcome, and co-morbidity. *Eur J Heart Fail* 2016; **18**: 490–9.

Senni M, Tribouilloy CM, Rodeheffer RJ, *et al.* Congestive heart failure in the community: trends in incidence and survival in a 10-year period. *Arch Intern Med* 1999; **159**: 29–34.

Shah RV, Rong J, Larson MG, *et al.* Associations of Circulating Extracellular RNAs With Myocardial Remodeling and Heart Failure. *JAMA Cardiol* 2018; **3**: 871.

Shah SA, Kambur T, Chan C, Herrington DM, Liu K, Shah SJ. Relation of Short-Term Heart Rate Variability to Incident Heart Failure (from the Multi-Ethnic Study of Atherosclerosis). *Am J Cardiol* 2013; **112**: 533–40.

Shahar E, Lee S, Kim J, Duval S, Barber C, Luepker RV. Hospitalized heart failure: rates and long-term mortality. *J Card Fail* 2004; **10**: 374–9.

Shan C, Chen Y, Ma Y, *et al.* [Incidence and distribution of chronic heart failure in the elderly from Xinjiang]. *Zhonghua Liu Xing Bing Xue Za Zhi Zhonghua Liuxingbingxue Zazhi* 2014; **35**: 1007–10.

Shiba N, Shimokawa H. Chronic heart failure in Japan: Implications of the CHART studies. *Vasc Health Risk Manag*; : 12.

Smeets M, Vaes B, Mamouris P, *et al.* Burden of heart failure in Flemish general practices: a registry-based study in the Intego database. *BMJ Open* 2019; **9**: e022972.

Sosin MD, Bhatia GS, Zarifis J, Davis RC, Lip GYH. An 8-year follow-up study of acute admissions with heart failure in a multiethnic population. *Eur J Heart Fail* 2004; **6**: 669–72.

Sree Chitra Tirunal Institute for Medical Sciences and Technology (SCTIMST) (India). India Sree Chitra Tirunal Institute for Medical Sciences and Technology Heart Failure Registry 2006. .

Sree Chitra Tirunal Institute for Medical Sciences and Technology (SCTIMST) (India). India Sree Chitra Tirunal Institute for Medical Sciences and Technology Heart Failure Registry 2001. .

Staszewsky L, Cortesi L, Tettamanti M, *et al.* Outcomes in patients hospitalized for heart failure and chronic obstructive pulmonary disease: differences in clinical profile and treatment between 2002 and 2009: Outcomes and treatments in heart failure with COPD. *Eur J Heart Fail* 2016; **18**: 840–8.

Störk S, Handrock R, Jacob J, *et al.* Epidemiology of heart failure in Germany: a retrospective database study. *Clin Res Cardiol* 2017; **106**: 913–22.

Taylor CJ, Harrison C, Britt H, Miller G, Hobbs FR. Heart Failure and Multimorbidity in Australian General Practice. *J Comorbidity* 2017; **7**: 44–9.

Tiller D, Russ M, Greiser KH, *et al.* Prevalence of Symptomatic Heart Failure with Reduced and with Normal Ejection Fraction in an Elderly General Population–The CARLA Study. *PLoS ONE* 2013; **8**: e59225.

Tribouilloy C, Buiciuc O, Rusinaru D, Malaquin D, Lévy F, Peltier M. Long-term outcome after a first episode of heart failure. A prospective 7-year study. *Int J Cardiol* 2010; **140**: 309–14.

Tsao CW, Lyass A, Enserro D, *et al.* Temporal Trends in the Incidence of and Mortality Associated With Heart Failure With Preserved and Reduced Ejection Fraction. *JACC Heart Fail* 2018; **6**: 678–85.

Tseng C-H. Clinical features of heart failure hospitalization in younger and elderly patients in Taiwan: HEART FAILURE HOSPITALIZATION IN TAIWAN. *Eur J Clin Invest* 2011; **41**: 597–604.

Tsuchihashi M, Tsutsui H, Kodama K, Kasagi F, Takeshita A. Clinical characteristics and prognosis of hospitalized patients with congestive heart failure--a study in Fukuoka, Japan. *Jpn Circ J* 2000; **64**: 953–9.

Tuppin P, Cuerq A, de Peretti C, *et al.* Two-year outcome of patients after a first hospitalization for heart failure: A national observational study. *Arch Cardiovasc Dis* 2014; **107**: 158–68.

Tuppin P, Rivière S, Rigault A, *et al.* Prevalence and economic burden of cardiovascular diseases in France in 2013 according to the national health insurance scheme database. *Arch Cardiovasc Dis* 2016; **109**: 399–411.

Uijl A, Koudstaal S, Vaartjes I, *et al.* Risk for Heart Failure. *JACC Heart Fail* 2019; **7**: 637–47.

van Jaarsveld CHM, Ranchor AV, Kempen GIJM, Coyne JC, van Veldhuisen DJ, Sanderman R. Epidemiology of heart failure in a community-based study of subjects aged ≥57 years: Incidence and long-term survival. *Eur J Heart Fail* 2006; **8**: 23–30.

Vanhercke D, Pardaens S, Weytjens C, *et al.* Prevalence, Determinants, and Prognostic Significance of Pulmonary Hypertension in Elderly Patients Admitted with Acute Decompensated Heart Failure: A Report from the BIO-HF Registry. *Echocardiography* 2015; **32**: 1333–8.

Wasywich CA, Gamble GD, Whalley GA, Doughty RN. Understanding changing patterns of survival and hospitalization for heart failure over two decades in New Zealand: utility of 'days alive and out of hospital' from epidemiological data. *Eur J Heart Fail* 2010; **12**: 462–8.

Zannad F, Briancon S, Juilliere Y, *et al.* Incidence, clinical and etiologic features, and outcomes of advanced chronic heart failure: the EPICAL study. *J Am Coll Cardiol* 1999; **33**: 734–42.

~~Zarrinkoub R, Wettermark B, Wändell P, *et al.* The epidemiology of heart failure, based on data for 2.1 million inhabitants in Sweden. *Eur J Heart Fail* 2013; **15**: 995–1002.~~

Zarrinkoub R, Wettermark B, Wändell P, *et al.* The epidemiology of heart failure, based on data for 2.1 million inhabitants in Sweden. *Eur J Heart Fail* 2013; **15**: 995–1002.

PubMed search string:

2017: "heart failure"[TIAB] AND (epidemiology[MeSH Terms] OR prevalence[TIAB] OR incidence[TIAB] OR mortality[TIAB]) AND ("1990/01/01"[PDAT] : "2016/09/02"[PDAT]) NOT “animal model” NOT rat NOT mice NOT diabetes[TIAB] NOT “renal transplant”[TIAB].

2020: "heart failure"[TIAB] OR “cardiac failure”[TIAB]) AND (epidemiology[MeSH Terms] OR prevalence[TIAB] OR incidence[TIAB] OR “excess mortality”[TIAB] OR “case fatality”[TIAB]) AND ("2016/01/01"[PDAT] : "2020/1/2"[PDAT]) NOT “animal model” NOT rat NOT mice NOT diabetes[TIAB] NOT “renal transplant”[TIAB]. 4,469 initial studies were returned and 27 sources were added.

Supplemental Tables

Table S1: Reported Prevalence of Heart Failure Point Estimates and Sample Sizes in 45 Studies Identified in Systematic Review.
When estimates were only reported in detailed age- or sex-categories (such as 10-year age groups or both sexes), we calculated effective sample sizes from reported standard error based on the Wilson Score Interval, and then collapsed cases and sample sizes to re-estimate a mean value for each year of the study.

Table S2: Reported Incidence of Heart Failure Point Estimates and Sample Sizes in 41 Studies Identified in Systematic Review.
When estimates were only reported in detailed age- or sex-categories (such as 10-year age groups or both sexes), we calculated effective sample sizes from reported standard error based on the Wilson Score Interval, and then collapsed cases and sample sizes to re-estimate a mean value for each year of the study.

Table S3: Reported One-Year Case Fatality of Heart Failure Point Estimates and Sample Sizes in 44 Studies Identified in Systematic Review.
When estimates were only reported in detailed age- or sex-categories (such as 10-year age groups or both sexes), we calculated effective sample sizes from reported standard error based on the Wilson Score Interval, and then collapsed cases and sample sizes to re-estimate a mean value for each year of the study.

Table S1: Reported Prevalence of Heart Failure Point Estimates and Sample Sizes in 45 Studies Identified in Systematic Review.

**Study**	**Prevalence, %**	**Sample Size**
Abhayaratna WP, 2006	6.28 (4.94-7.63)	1273
Agarwal AK, 2001	0.52 (0.49-0.55)	225000
Alehagen U, 2009	11.2 (9.11-13.29)	886
Ammar KA, 2007	2.41 (1.74-3.09)	2029
Anguita Sánchez M, 2008	6.06 (4.77-7.36)	1322
Azevedo A, 2006	7.66 (5.52-9.79)	609
Carmona M, 2011	0.95 (0.4-1.5)	1279
Ceia F, 2002	10.49 (9.73-11.25)	6300
Ceia F, 2005	6.51 (4.79-8.23)	805
Cho H, 2018	1.17 (1.16-1.19)	1727471
Cortina A, 2001	5.12 (2.88-7.35)	391
Cuthbert JJ, 2019	1.01 (0.81-1.22)	9390
Danielsen R, 2017	3.63 (3.14-4.11)	5706
Davies M, 2001	2.32 (1.85-2.79)	3960
Di Bari M, 2004	9.11 (6.67-11.54)	549
Dongfeng G, 2003	0.87 (0.72-1.01)	15518
Einarsson H, 2017	3.6 (2.93-4.28)	2961
Engelfriet PM, 2011	1.71 (1.59-1.82)	49517
Hao G, 2019	1.41 (0.67-2.15)	1040
Hung YT, 2000	0.23 (0.22-0.24)	744243
Jiménez-García R, 2014	0.65 (0.64-0.66)	6200057
Kannel WB, 1991	4.38 (3.73-5.03)	3819
Khan H, 2014	16.8 (15.44-18.15)	2935
Khera R, 2017	17.66 (17.64-17.68)	12749680
Kitzman DW, 2001	8.57 (7.98-9.17)	8473
Knox SA, 2008	1.8 (1.5-2.1)	7545
Kupari M, 1997	8.18 (5.75-10.61)	501
Lee H, 2016	0.88 (0.87-0.88)	38307984
Leibowitz D, 2011	12 (8.97-15.03)	450
Leibowitz D, 2019	15.08 (8.65-21.51)	126
Lindmark K, 2019	1.63 (1.62-1.63)	37792514
McAlister FA, 2004	0.71 (0.68-0.74)	307741
McSwain M, 1999	2.55 (1.83-3.27)	1887
Mosterd A, 1999	3.45 (2.97-3.94)	5540
Mureddu GF, 2012	6.75 (5.61-7.88)	1881
Murphy NF, 2004	1.5 (1.43-1.57)	114788
Piccinni C, 2017	3.74 (3.68-3.81)	313787
Redfield MM, 2003	2.2 (1.56-2.85)	2042
Shan C, 2014	4.3 (3.66-4.95)	3857
Smeets M, 2019	1.98 (1.96-2)	2559128

Stork S, 2017	3.9 (3.88-3.92)	3177564
Taylor CJ, 2017	0.99 (0.98-1)	2593510
Tiller D, 2013	9.66 (8.24-11.07)	1688
Tuppin P, 2016	1.09 (1.08-1.09)	48514321
Zarrinkoub R, 2013	3.57 (3.55-3.58)	4625034

S1: Reported Prevalence of Heart Failure Point Estimates and Sample Sizes in 45 Studies Identified in Systematic Review [GAR1]

Table S2: Reported Incidence of Heart Failure Point Estimates and Sample Sizes in 41 Studies Identified in Systematic Review

**Study**	**Incidence, per 100**	**Sample Size (in Person-Years)**
Al Suwaidi J, 2004	0.14 (0.12-0.15)	134815
Barasa A, 2014	0.72 (0.7-0.75)	443995
Bleumink GS, 2004	1.44 (1.34-1.55)	50269
Borne Y, 2014	0.19 (0.18-0.21)	368053
Conrad N, 2018	0.52 (0.51-0.52)	24877519
Corrao G, 2014	0.73 (0.72-0.74)	3597225
Curtis LH, 2008	4.33 (4.3-4.36)	2070015
Einarsson H, 2017	0.17 (0.15-0.19)	188644
Fox KF, 2001	0.48 (0.46-0.5)	364792
Hinton W, 2018	0.12 (0.12-0.13)	5249994
Hung YT, 2000	0.67 (0.65-0.69)	676997
Huusko J, 2019	0.23 (0.15-0.31)	15594
Kannel WB, 1991	1.98 (1.91-2.04)	167280
Khan H, 2014	1.81 (1.65-1.97)	2935
Khera R, 2017	3.27 (3.26-3.28)	12749680
Lee DS, 2004	2.97 (2.97-2.98)	87491152
Li R, 2019	0.93 (0.84-1.02)	43204
Lindmark K, 2019	0.96 (0.96-0.97)	56268824
Loehr LR, 2008	0.65 (0.61-0.68)	198422
Magnussen C, 2019	0.52 (0.5-0.53)	999833
McAlister FA, 2004	0.2 (0.18-0.22)	307741
Murphy NF, 2004	0.87 (0.83-0.9)	307436
Nayor M, 2016	0.49 (0.42-0.56)	38216
Ohlmeier C, 2015	1.11 (1.07-1.15)	247252
Piccinni C, 2017	0.59 (0.56-0.61)	338506
Rautiainen S, 2013	0.2 (0.18-0.21)	394059
Remes J, 1992	0.26 (0.19-0.33)	23034
Rywik S, 1999	0.44 (0.23-0.66)	4013
Sangaralingham LR, 2016	0.67 (0.67-0.67)	16360058
Senni M, 1999	2.4 (2.33-2.48)	147938
Shah RV, 2018	0.56 (0.27-0.85)	2681
Shah SA, 2013	0.27 (0.21-0.33)	33013
Stork S, 2017	0.65 (0.65-0.66)	4033768
Tsao CW, 2018	2.18 (2.1-2.27)	115703
Tseng C-H, 2011	0.36 (0.35-0.38)	741054
Uijl A, 2019	0.12 (0.11-0.13)	569362
Wasywich CA, 2010	0.19 (0.18-0.19)	80000000

Zannad F, 1999	0.1 (0.1-0.11)	1592263
Zarrinkoub R, 2013	0.66 (0.66-0.67)	4625034
de Giuli F, 2005	3.4 (3.4-3.4)	72608310
van Jaarsveld CHM, 2006	1.28 (1.14-1.42)	25662

~~Table S2: Reported Incidence of Heart Failure Point Estimates and Sample Sizes in 41 Studies Identified in Systematic Review~~

Table S3: Reported One-Year Case Fatality of Heart Failure Point Estimates and Sample Sizes in 44 Studies Identified in Systematic Review

**Study**	**Mortality at 1 year, %**	**Sample Size**
AHRI, 2013	30.79 (28.18-33.4)	1205
Alexander M, 1999	32.2 (31.9-32.5)	90316
Ammar KA, 2007	4.08 (1.13-13.71)	49
Amsalem Y, 2008	28.82 (27.38-30.27)	3792
Atzema CL, 2015	14.77 (14.58-14.97)	125691
Barasa A, 2014	30.51 (30.37-30.64)	443995
Berkovitch A, 2015	30.02 (28.11-31.93)	2212
Bleumink GS, 2004	37 (33.48-40.52)	725
Chamberlain AM, 2013	9.66 (6.53-12.79)	352
Chen J, 2011	31.28 (31.24-31.32)	4866309
Coles AH, 2015	44.75 (42.9-46.6)	2780
Corrao G, 2014	29.36 (28.71-30.02)	18795
Cowie MR, 2000	38 (31.53-44.47)	220
Ezekowitz JA, 2011	17.02 (16.75-17.3)	72043
Gamble J-M, 2011	16.9 (16.62-17.17)	72043
Gioli-Pereira L, 2019	6.76 (4.88-8.65)	695
Goda A, 2010	4.36 (2.69-6.02)	597
Hai J-J, 2016	14.08 (11.86-16.29)	952
Heller RF, 2000	28.65 (25.65-31.65)	877
Hoekstra T, 2013	36.36 (31.53-41.19)	385
Kaplon-Cieslicka A, 2016	19.68 (16.53-22.82)	620
Lassus JPE, 2013	27.1 (23.58-30.61)	620
Lee DS, 2004	35.71 (35.39-36.02)	88440
Lyu S, 2019	7.02 (5.18-8.86)	755
Maison P, 2013	20.64 (15.86-25.42)	281
Makubi A, 2016	23.6 (19.47-27.73)	411
McAlister FA, 2013	33.27 (32.77-33.77)	34369
McManus DD, 2013	34.66 (33.72-35.61)	9748
Nakano A, 2013	15.11 (14.16-16.06)	5433
Nakano A, 2016	17.11 (16.64-17.59)	24301
Novack V, 2010	28.68 (27.7-29.66)	8246
Ohlmeier C, 2015	33.69 (33.41-33.97)	109363
Oster HS, 2013	29.16 (27.31-31.01)	2332
Ozieranski K, 2016	13.8 (10.99-16.61)	587
Parenica J, 2013	20.3 (18.95-21.65)	3438
Rathore SS, 2006	35.6 (35.01-36.19)	25086
SCTIMST, 2001	35.84 (32.32-39.36)	717
SCTIMST, 2006	30.53 (27.31-33.76)	786

Sartipy U, 2014	20.12 (19.77-20.47)	51043
Schmidt M, 2016	38.98 (38.79-39.16)	266692
Sosin MD, 2004	45.06 (38.62-51.51)	233
Staszewsky L, 2016	16 (15.4-16.61)	14111
Tuppin P, 2014	29.22 (28.89-29.56)	69958
Vanhercke D, 2015	26.93 (22.56-31.3)	401

~~Table S3: Reported One-Year Case Fatality of Heart Failure Point Estimates and Sample Sizes in 44 Studies Identified in Systematic Review~~

Supplemental Figures

Figure S1: PRISMA diagram
Caption: Flowchart of studies identified in systematic review.

Figure S2: Countries with Studies Reporting Estimates of Heart Failure Prevalence or Incidence
Caption: Countries with studies reporting estimates of HF prevalence or incidence. Red represents countries with studies reporting both prevalence and incidence; blue represents countries with only studies reporting incidence; and green represents countries with studies only reporting prevalence. White represents countries with no studies reporting estimates of prevalence or incidence.

Figure S3: Countries with Studies Reporting Estimates of Heart Failure Mortality
Caption: Countries with studies reporting estimates of HF mortality. White represents countries with no studies reporting estimates of mortality.

Figure S4: Reported Prevalence of HF in 45 Studies Identified in Systematic Review by Year
Caption: Prevalence of heart failure (%, or per 100) reported in 45 studies by the mid-year of the data, colored according to study, and arranged by demographic profile. Multi-year studies are shown as same-colored and same-shape points connected by a line. “All adults” refers to studies restricted to patients 18+; “Older adults” refers to studies restricted to patients 50+; “All ages” refers to studies including patients of all ages. When estimates were only available in detailed age- or sex-categories (such as 10-year age groups or both sexes), we calculated effective sample sizes from reported standard error based on the Wilson Score Interval, and then collapsed cases and sample sizes to re-estimate a mean value for each year of the study.

Fi-gure S5: Reported Incidence of HF in 41 Studies Identified in Systematic Review by Year
Caption: Incidence of heart failure (per 100 person-years) reported in 41 studies by the mid-year of the data, colored according to study, and arranged by demographic profile. Multi-year studies are shown as same-colored and same-shape points connected by a line. “All adults” refers to studies restricted to patients 18+; “Older adults” refers to studies restricted to patients 50+; “All ages” refers to studies including patients of all ages. When estimates were only available in detailed age- or sex-categories (such as 10-year age groups or both sexes), we calculated effective sample sizes from reported standard error based on the Wilson Score Interval, and then collapsed cases and sample sizes to re-estimate a mean value for each year of the study.

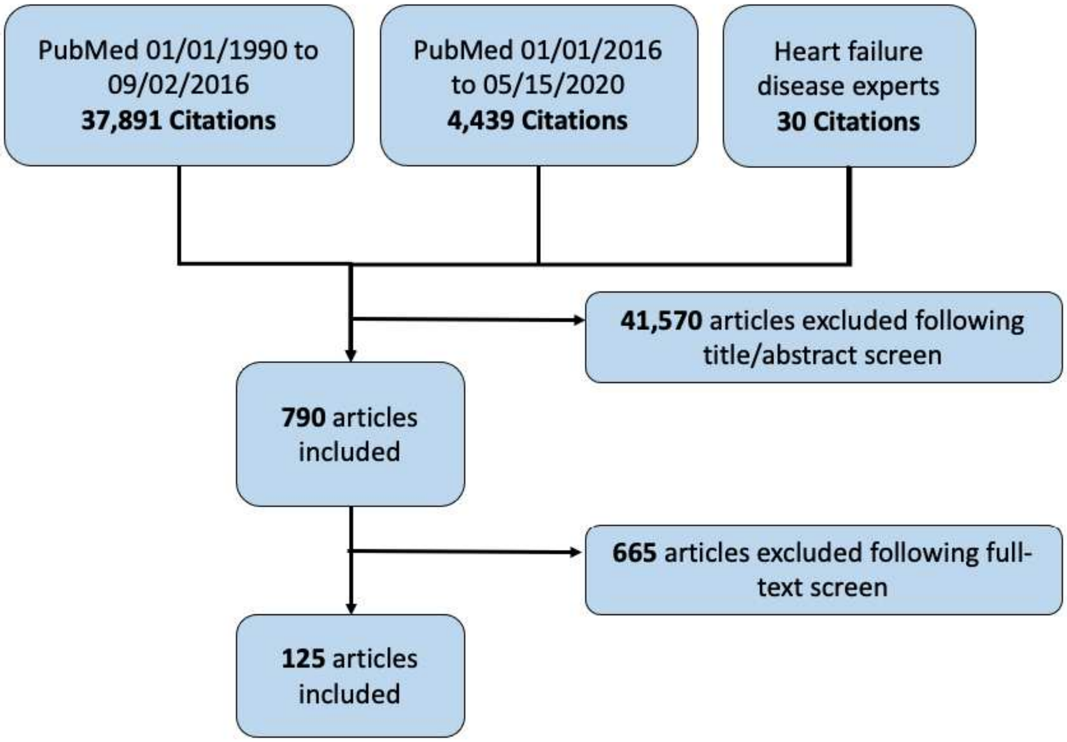

Figure S1: PRISMA diagram

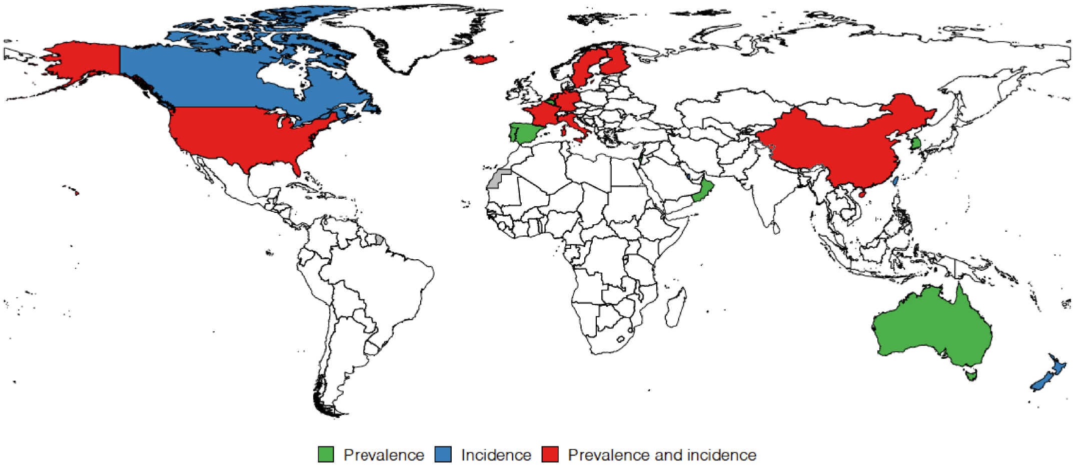

Figure S2: Countries with Studies Reporting Estimates of Heart Failure Prevalence or Incidence

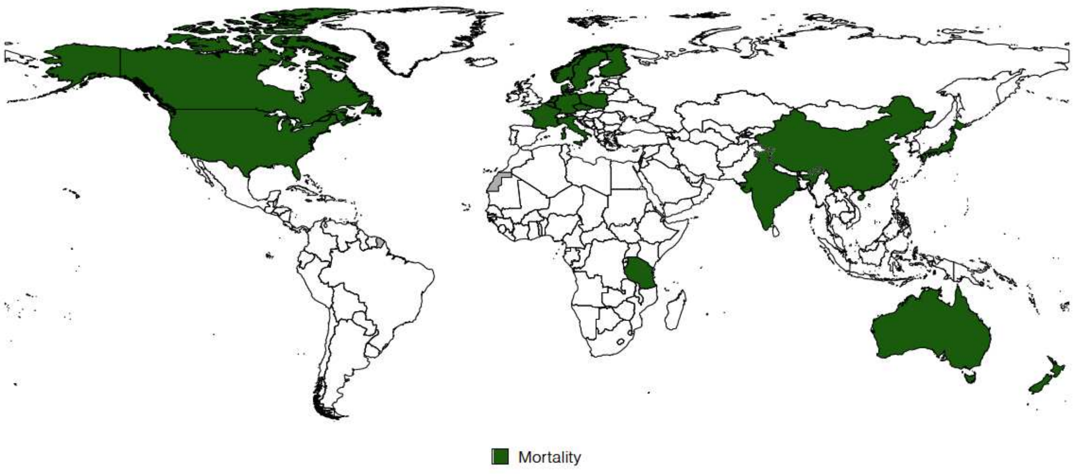

Figure S3: Countries with Studies Reporting Estimates of Heart Failure Mortality

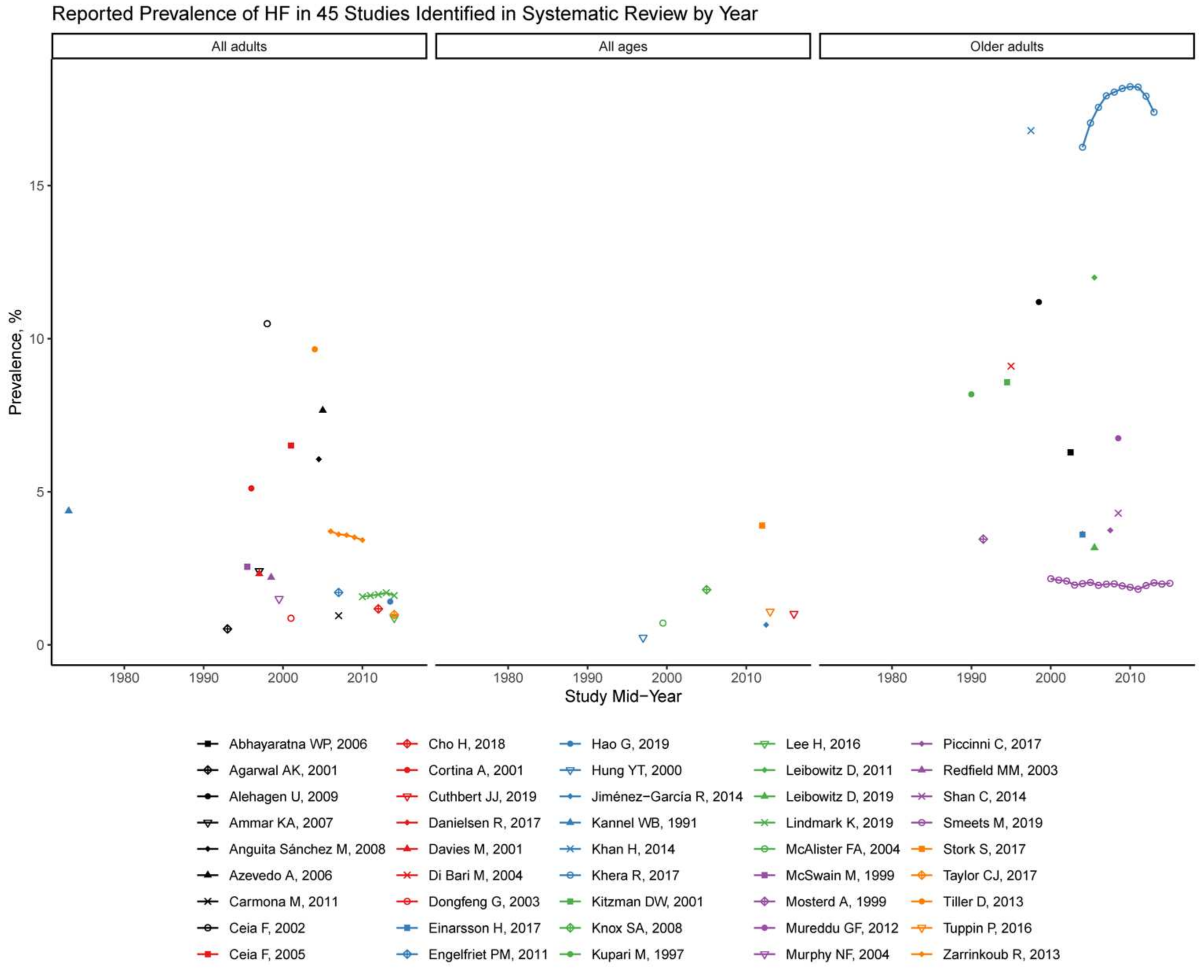

Figure S4: Reported Prevalence of HF in 45 Studies Identified in Systematic Review by Year

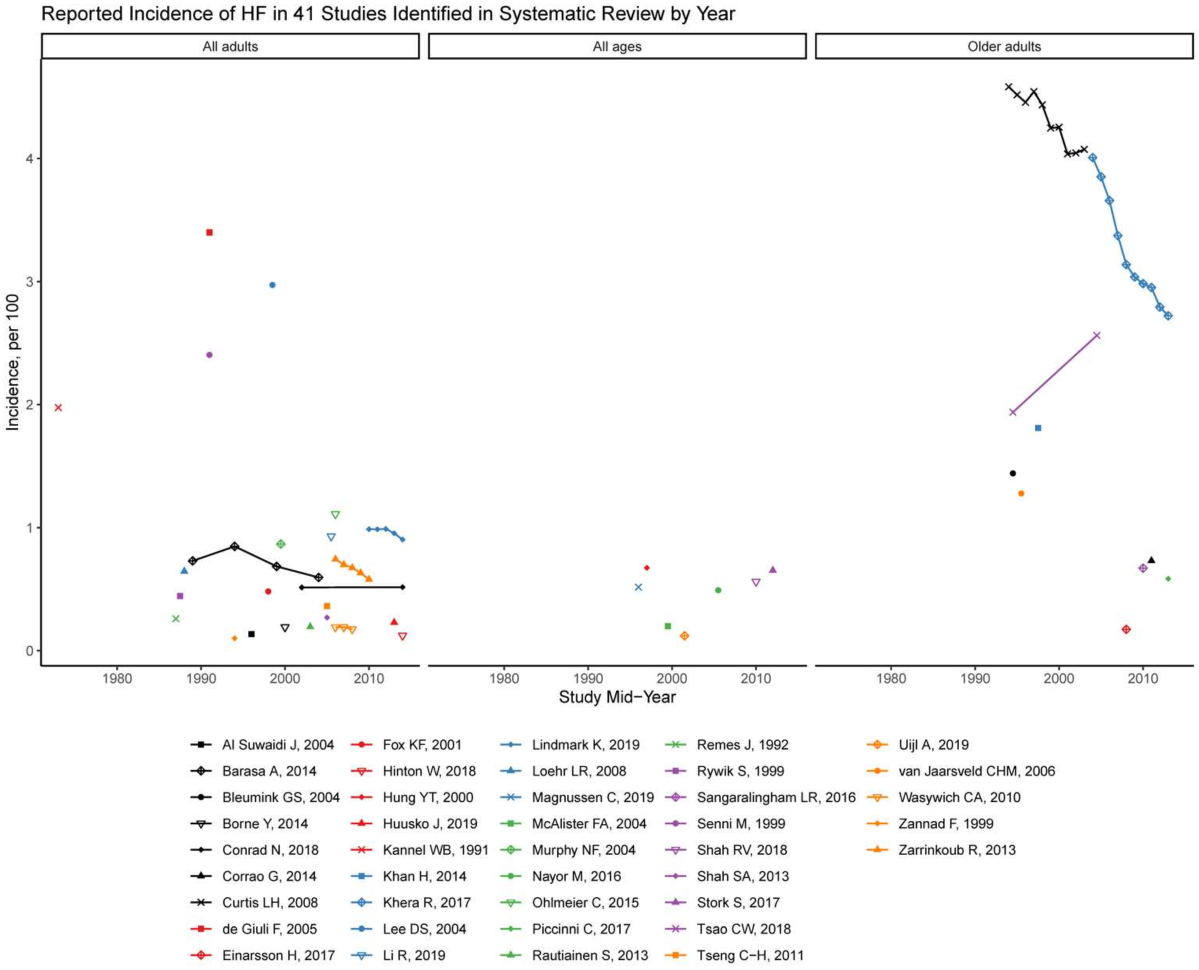

Figure S5: Reported Incidence of HF in 41 Studies Identified in Systematic Review by Year
